# Gall Bladder Surgery—Shall We Drain or Remove*Read at the Medical Association of Central New York, Buffalo, Oct. 19, 1916.

**Published:** 1917-04

**Authors:** Charles W. Hennington

**Affiliations:** Rochester


᾿# BUFFALO MEDICAL JOURNAL

Yearly Volume 72 APRIL, 1917 Number 9

### ORIGINAL ARTICLES

The right is reserved to decline papers not dealing with practical medical and surgical subjects, and such as might offend or fail to interest readers. Contributors are solely responsible for opinions, methods of expression and revision of proof.

### Gall Bladder Surgery—Shall We Drain or Remove.*

DR. CHARLES W. HENNINGTON, Rochester.

Very likely many of those present will attend the Clinical Congress of Surgeons to be held at Philadelphia next week. It is noteworthy that one entire evening will be given over to a discussion of the subject here proposed. The principle speakers are: Finney of Baltimore, Charles Mayo of Rochester, Fred Lund of Boston, and Da Costa and Deaver of Philadelphia.

A large number of articles upon gall bladder surgery have been appearing in the various medical journals of late. It is significant, therefore, that this subject of the relative merits of drainage or removal should have been given so important a place upon the program. All this indicates to me that there is still the greatest diversity of opinion as to which is the operation of choice in given classes of cases. That our practice will soon crystallize seems to me very likely, and this also adds to the importance of the discussion going on at the present time.

Since gall bladder surgery began, these two operations have been recognized. From the historical point of view, mere drainage of the abscess was, of course, the earliest operation. In fact nature herself not infrequently performed this obvious measure unassisted. Sometimes the perforation was external but more often it occurred into some neighboring organ, most often the intestines. Yet very early after surgical intervention began the removal of the gall bladder was proposed and carried out. Soon, however, the drainage operation, because of its safety, was recognized as the one of

*Read at the Medical Association of Central New York, Buffalo, Oct. 19, 1916.

choice, and continued so for many years. It is only of late that cholecystectomy has again come to the front. The larger number of articles favor cholecystectomy. A not inconsiderable number however continue defending the merits of cholecystostomy as the standard method in the largest number of cases. The practice in the various clinics throughout the country is far from standardized.

Recently, in order to throw further light upon this subject, Donald Guthrie of Sayre, Pennsylvania, sent out a questionaire of seven very well selected points to the various prominent surgeons, and received 45 replies. He presented the results at a meeting of the Section in Obstetrics Gynecology and Abdominal Surgery at the Detroit meeting of the A. M. A. Subsequently the paper was published in the Journal of the A. M. A., Aug. 26, 1916.

As his study referred more particularly to the leaders of the profession, I thought it would be of interest and value to discover what the feeling was among the average practitioners of surgery. With this object in mind, I so arranged a program for the Surgical Section of the Rochester Academy of Medicine, of which I happen to be the chairman. Of course it is only too evident that what is the established and successful practice of a clinic, such as that of the Mayos at Rochester, Minn., may not be entirely feasible and successful, as practiced by the various operators in the various hospitals in a city such as Rochester, N. Y.

So at our meeting, held last week, we used the same seven questions propounded by Guthrie. The various men on the program were asked to discuss them from their own point of view, as a result of their own experience. These various papers, or talks, were all concise and to the point. As the seven questions had been written upon a large blackboard, placed before the meeting, there was an animated discussion. Thereupon, in order to complete and sum up the discussion, Dr. Guthrie had been invited to come and personally give us the results gathered from his studies of the opinion of 45 leading American surgeons. I believe the meeting proved a profitable exercise for all there present. In fact there were some points brought out which I consider of sufficient value to venture to bring before you here.

The seven questions were:

1. What percentage of cases of cholecystostomy have had a recurrence of trouble?

2. Are you performing cholecystectomy more frequently than in the past?

3. Have the results been better than when simple drainage was used?

4. In what cases do you consider cholecystectomy the operation of choice?

5. What are the contra-indications against its employment?

6. As a rule, how do you treat empyema of the gall bladder?

7. How does the mortality of the two operations compare?

(1). A definite percentage of recurrence after cholecystostomy seems inherent and unavoidable. Guthrie concludes from the study of the replies he received, that the actual percentage of recurrence is probably about 8% or 9%. It must be recognized also that cholecystectomy, even in favorable cases, is not entirely free from subsequent trouble. One of our local men urged that more prolonged drainage after operation might actually cure many a gall bladder, which another operator might deem necessary to remove. Another (myself in this case) brought out the point that recurrence was not necessarily to be charged to the operation itself, but due to some inherent tendency of the patient toward the formation of gall stones or biliary sand. That these recurrent attacks, like the preceding ones, were due to a hypercholesterinaemia dependent upon faulty metabolism. That by making the analyses of the blood and giving strict attention to diet, the re-formation of stones could be avoided. This refers to Aschoff's theory of gall stone formation. These individuals retain their lipoids and the bile becomes saturated with cholesterin, leading to a sudden precipitation of stones. Only when stones on sectioning show concentric layers of deposit should the cause be assigned to inflammatory processes of the gall bladder. After operation a physiological chemist should make repeated examinations of the blood (only 2 c.c. from the ear is required) to determine the percentage of cholesterin. Guided by these tests the diet of eggs, cream, butter, all fats, meat and fish should be reduced. Only vegetables, cereals, sugar and skimmed milk being allowed in abundance.

(2). Cholecystectomy is being done more frequently than in the past, but evidently the proportion compared with cholecystostomy is small, and rightly so. It was thought that the Mayo proportion in 1915 of 915 ectomies to only 60 ostomies, was never applicable to a community like Rochester, N. Y., because the nature of the cases was very different. This was discussed in detail. Suffice it to say, that Mayo conclusions, as to such a matter are not necessarily absolutely applicable to another series of cases, occuring normally to any one community. The recognition of the gall bladder as a focus of infection leading to infections elsewhere than in its immediate vicinity, and leading to degenerative changes in other important organs, would strongly favor removal in many instances.

(3). Whether the results have been better with removal than drainage really depends upon whether the correct indications or contra-indications were observed. That group of gall bladder cases in which some operators do one operation and others do the other operation should be carefully studied from large series of cases in which one or the other operation was done under otherwise relatively similar conditions. Only in this way can the relative merit of the two operations be determined. This difficulty of finding an exact and safe basis for comparison would seem obvious, but evidently is often overlooked by advocates of one or another method of treatment.

(4). Cholecystectomy should be considered indicated in disease of the gall bladder wall and in damage to the cystic duct. These chronic thick-walled gall bladders may be either small or large, and with or without stones. Early cancer limited to the gall bladder and gangrene of the gall bladder are definite indications. The damage to the cystic duct may be either actual stricture or potential stricture, due to injury by a stone of such a degree as to make subsequent stricture probable. This indication is sometimes stated as cystic gall bladder or hydrops of the gall bladder, but obviously the real lesion is obstruction of the cystic duct. An occasional indication for removal is found in extensive perforation or laceration.

(5). The contra-indications against cholecystectomy are associated pancreatic disease, associated disease of common duct involving its patency, and associated disease of the smaller bile passages. Pancreatic disease must be treated by extremely prolonged drainage of the gall bladder. The patency of the common duct must be established before removal of the gall bladder by passing a probe downward into the duodenum. A general cholangitis is often difficult to determine and is a source of anxiety and annoyance.

Many conservative operators class acute empyema as a definite contra-indication. Not all however.

Certain **technical** difficulties are contra-indications to cholecystectomy, such as great obesity of patient or perihepatic adhesions, both making good exposure impossible. Oedema of gastro-hepatic omentum with inability to recognize the ducts is a contra-indication of this type.

Further contra-indications are **inexperience** of the operator and especially of the anaesthetist; one who may not appreciate that manipulations about the liver may cause straining and coughing even in deep anaesthesia.

Finally desperate condition of the patient forms an absolute contra-indication.

(6). The treatment of empyema of the gall bladder offers diverse views. As a rule, however, cholecystostomy is preferred. In fact in Guthrie's answers, 34 out of 44 favor it. Some so strongly that they consider acute empyema a contraindication to cholecystectomy. Without any doubt, however, a removal may often be done in individual instances. It seems safer to attempt it in chronic empyemas. A two-stage operation solves the difficulty, namely, first drainage then removal.

(7). As to the comparative mortality of the two operations. With very experienced operators, at their own well conducted clinics, the difference in mortality is very slight, probably below 2%, for either operation. But with the operator of more limited experience, and more limited advantages as to assistance, facilities, etc., cholecystectomy may have a very high mortality. The sources of dangers are shock, haemorrhage from cystic artery, or from cut liver surface, and damage to common or hepatic ducts.

**Conclusion.** Thus have I tried to summarize as much as possible. I hope that I do not give the impression that there were no differences of opinion, for on the contrary, such diversity was evident on each point. My endeavor has been to sum up in a constructive way. Unavoidably my own opinions could not be suppressed in this effort, and so I crave your indulgence..

633 Park Ave.

---

**Shell Bullet Enveloped in Great Omentum and Movable in Hernial Sac.** L.-C. Bailleul, Le Prog. Med., Dec. 1916. (The non-surgical history of the case is so inspiring that we make no apologies for including it in the abstract). Albert, a colonial soldier, aged 36, was wounded Aug. 30, 1914, about 14½ o'clock, in the right infrascapular region. He continued firing for a quarter of an hour, then went to a neighboring house to have the wound washed. He left this shelter to carry a wounded comrade to a place of shelter. At 17½ o'clock, he was captured by the Germans but escaped half an hour later and reported to an ambulance station. He recovered rapidly, without peritoneal reaction and rejoined his depot at Marseilles Oct. 3, soon returning to the front. He served till Jan. 9 when he noticed a left inguinal hernia. Radical operation was performed Jan. 30, during which the ball was found surrounded by fringes of great omentum. He was discharged after an uneventful convalescence, Feb. 22.

